# Supplementary material for: Comparative multiomics analysis of cell physiological state after culture in a basket bioreactor
Source: Sci Rep. 2022 Nov 23;12:20161. doi: 10.1038/s41598-022-24687-4 (PMC9686226; doi:10.1038/s41598-022-24687-4)
Supplement: Supplementary file 1 — Supplementary Information 1. [file 41598_2022_24687_MOESM1_ESM.zip › raw data/Proteomic raw data/6.KEGGenrichment/EG.vs.CG/UP/pathway/src/04979.html]

map04979


- K13443

- Up regulated proteins

A0A0D9RJC1(Description:Epididymal secretory protein E1;Gene:NPC2;log2FC:1.2660)

- K05664

- Up regulated proteins

A0A0D9RRV8(Description:Bile salt export pump;Gene:ABCB11;log2FC:1.0388)

Close
